# Supplementary material for: Prognostic value of baseline carotid blood flow in critically ill children with septic shock
Source: PLoS One. 2021 Jul 23;16(7):e0251154. doi: 10.1371/journal.pone.0251154 (PMC8302249; doi:10.1371/journal.pone.0251154)
Supplement: Attachment — Submitted filename: response to reviewers.docx [file pone.0251154.s002.docx]

Response to reviewers

Dear Sir,

It was great pleasure to allow me to improve the manuscript.

Please ensure that your manuscript meets PLOS ONE's style requirements, including those for file naming. The PLOS ONE style templates :

Reformatting according to PLOS ONE requirements.

2. We noticed you have some minor occurrence of overlapping text with the following previous publications, which needs to be addressed:

https://rd.springer.com/article/10.1007/s40477-014-0139-9?code=897273d4-47ee-4cff-8464-94a130407a9e
https://westjem.com/videos/can-emergency-physicians-perform-common-carotid-doppler-flow-measurements-to-assess-volume-responsiveness.html
<https://theultrasoundjournal.springeropen.com/articles/10.1186/s13089-017-0065-0>

Introduction was modified to avoid the overlapping text with previous publications to

3. Please ensure you have discussed any potential limitations of your study in the Discussion, including study design, sample size and/or potential confounders.

Study limitations section was added. Lines 188-191

first, our study is a single center , with small number of septic shock patients. Second, we did not observe if the changes in the cerebral autoregulation in pediatric septic shock could impact the carotid blood flow. Also, whether targeting increase carotid blood flow will improve survival or not needs further study.

4.Please provide a sample size and power calculation in the Methods, or discuss the reasons for not performing one before study initiation.

Considering the pilot nature of the study, we did not perform a priori sample size calculation. The number of patients included in the study was limited by logistic issues. Lines 85-86

4.Please provide additional details regarding participant consent.In the ethics statement in the Methods and online submission information, please ensure that you have specified what type you obtained (for instance, written or verbal, and if verbal, how it was documented and witnessed).

The study protocol was approved by Cairo university faculty of medicine ethical committee with written consent with approval number "I-071017. Line 52-53

5.Please include a separate caption for each figure in your manuscript.

Attached after the corresponding paragraph. Line 124

Line 142.

Reviewer #2: The authors evaluated the value of carotid artery ultrasound analysis in prediction of mortality in paediatric patients with septic shock.
Specific comments:
- The changes in CBF reported in this analysis might simply reflect the blood flow redistribution typical of septic shock. In fact, the changes were larger in cold than in warm shock. Hence, the reduction of CBF might be a feature of more advanced shock. The authors should comment on this: what is their view on this point? What is the potential clinical usefulness of the index? Should it just reflect advanced disease, would it have an actual impact on patient management?

Lines 180-187: The Surviving Sepsis Campaign International Guidelines for the Management of Septic Shock in Children 2020 reported that distinction between cold and warm shock if advanced hemodynamic monitoring is available may be helpful to assess patient physiology and direct inotropes and vasopressor therapy. We could observe that the carotid blood flow is less in patients with cold compared to warm shock type. However, the higher SVRI in cold shock could explain the reduction of carotid blood flow and whether that fluid augmentation and aiming at normalization of SVRI could influence the outcome needs to be explored.
- Which is the additive prognostic value of CBF over the traditional clinical parameters of hemodynamic impairment and/or hypo-perfusion of peripheral organs and tissues (lactate, indices of multi-organ failure, CVP)?

Line 167-168: The use point-of-care ultrasound to estimate the carotid blood flow is a rapid, easy to learn and noninvasive method for prognostic evaluation in critically unstable children who may not tolerate any invasive maneuver.
- ROC Curve in Figure 2 does not match with results provided in Results Section [AUC (95% CI): 0.3 (0.11 – 0.48), p=0.035]. Please check.

Line 140: [AUC (95% CI): 0.71 (0.52 – 0.89), p=0.035], sensitivity:71.4%, specifity:53.8%

- In the Results Section the authors stated: “Correlation analysis revealed significant correlation between CBF and age (r=0.42, p=0.008), weight (r=0.38, p=0.017), surface area (r=0.38, p=0.017) and SV (r=0.49, p=0.001)”. Correlations are indeed significant, but modest according to the “r” coefficients.

Line 126: Correlation analysis revealed modest significant correlation between CBF and age (r=0.42, p=0.008
