# Supplementary material for: Prognostic value of baseline carotid blood flow in critically ill children with septic shock
Source: PLoS One. 2021 Jul 23;16(7):e0251154. doi: 10.1371/journal.pone.0251154 (PMC8302249; doi:10.1371/journal.pone.0251154)
Supplement: Attachment — Submitted filename: response to reviewers.docx [file pone.0251154.s003.docx]

Response to reviewers

Dear Sir,

It was great pleasure to allow me to improve the manuscript.

Please review your reference list to ensure that it is complete and correct. If you have cited papers that have been retracted, please include the rationale for doing so in the manuscript text, or remove these references and replace them with relevant current references. Any changes to the reference list should be mentioned in the rebuttal letter that accompanies your revised manuscript. If you need to cite a retracted article, indicate the article’s retracted status in the References list and also include a citation and full reference for the retraction notice.

Reference list was revised and non of the papers were retracted

Reference 12 was missing and I added it to the reference section.

Reviewer #2: I am happy with the changes and the authors' response.
Some minor points still to address:
- Can you please provide inter and intra-observer reproducibilty data of CBF?
- Please provide a figure showing different patterns of CBF in different groups.

Studies were performed on ten healthy children before start of the study to evaluate interobserver and intraobserver reproducibility, the radiologist and the intensivist blinded to each other's results alternately performed two measurements on each patient. Intraobserver reproducibility was assessed between the observations by same observer. The intensivist performed the carotid flow scan and all the stored images were reviewed by the expert radiologist.

Figure 1 about subgroups:
